# Supplementary material for: Spatial transcriptomics reveal topological immune landscapes of Asian head and neck angiosarcoma
Source: Commun Biol. 2023 Apr 27;6:461. doi: 10.1038/s42003-023-04856-5 (PMC10140281; doi:10.1038/s42003-023-04856-5)
Supplement: Supplementary file 2 — Supplementary Information [file 42003_2023_4856_MOESM2_ESM.pdf]

SUPPLEMENTARY INFORMATION

Supplement to: Spatial transcriptomics reveal topological immune landscapes of Asian head and neck angiosarcoma

CONTENTS

1.0 Supplementary Figures

Supplementary Figure 1. Somatic mutational landscape of angiosarcomas from an Asian cohort.

Supplementary Figure 2. Distinct clusters of Asian angiosarcomas defined by immune microenvironmental and tumor-related pathways.

Supplementary Figure 3. Dot plot of spatial datasets showing gene expression patterns of cell type-specific markers.

2.0 Supplementary Data (excel file)

Supplementary Data 1. Clinical characteristics of patients with angiosarcoma in the study cohort

Supplementary Data 2. List of nonsynonymous somatic mutations identified from whole-genome sequencing of angiosarcomas

Supplementary Data 3. NanoString pathway scores (whole cohort)

Supplementary Data 4. NanoString cell type scores (log2, whole cohort)

Supplementary Data 5. Source data for Figure 6b-d

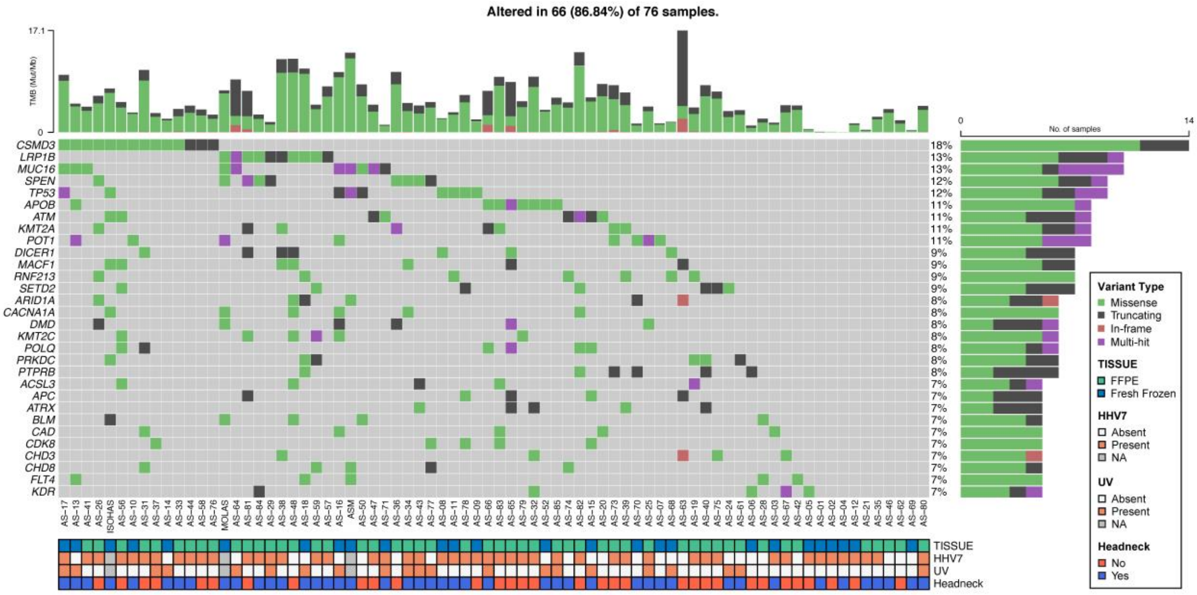

Supplementary Figure 1. Somatic mutational landscape of angiosarcomas from an Asian cohort. Oncoplot of somatic nonsynonymous variants of interest, including recurrent mutations in *CSMD3* (18%), *LRP1B* (13%), *MUC16* (13%), *SPEN* (12%), and *TP53* (12%). The median tumor mutation burden (TMB) was 4.50 mutations per coding megabase (mt/Mb) (range, 0.03 – 17.1 mt/Mb).

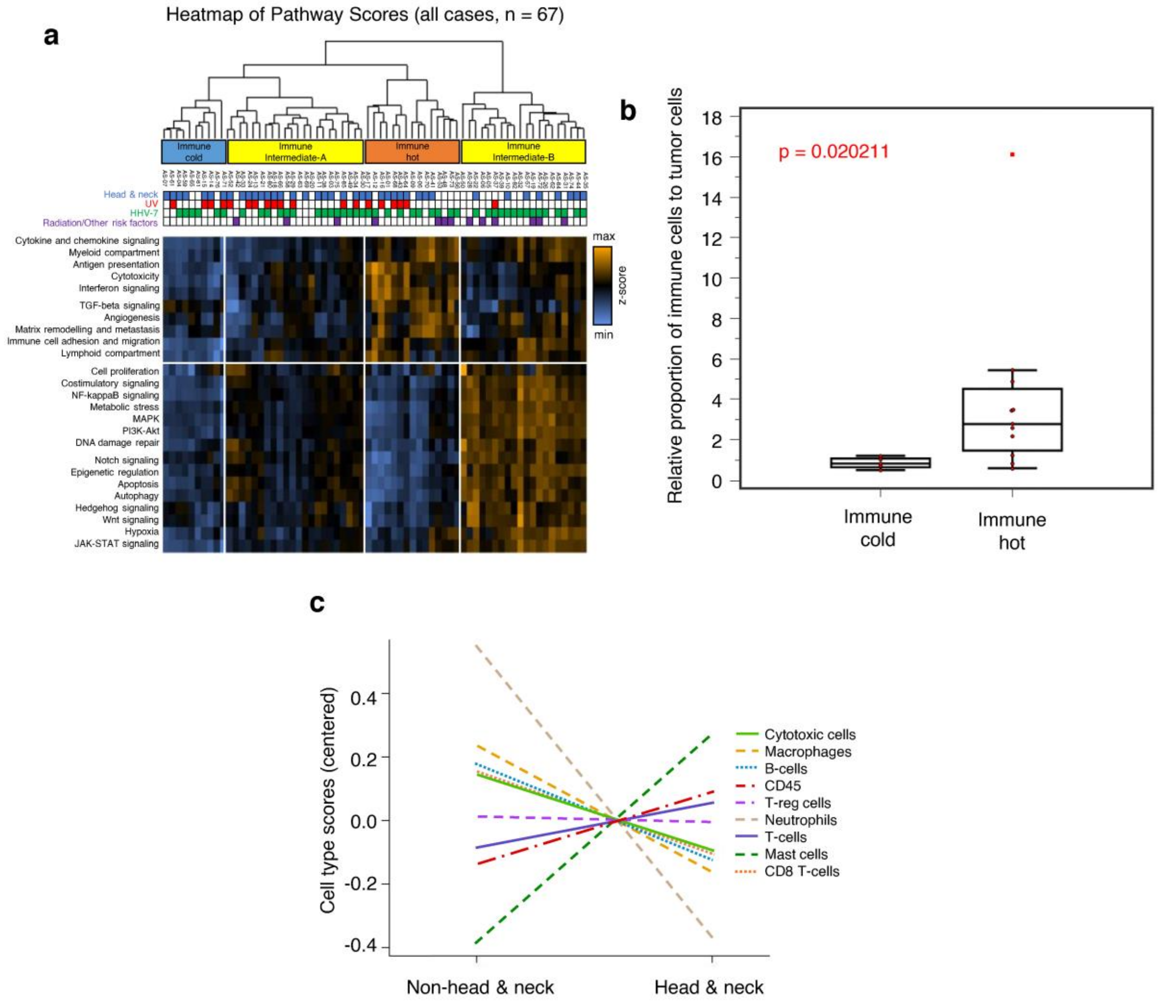

Supplementary Figure 2. Distinct clusters of Asian angiosarcomas defined by immune microenvironmental and tumor-related pathways. (a) NanoString IO360 panel profiling revealed clusters with distinct levels of immune signaling. Similar to the AS-HN results, the immune-hot cluster was characterized by relative upregulation of several immune-related pathways. The immune-intermediate cluster exhibited upregulation of several oncogenic signaling pathways, particularly in intermediate-B over intermediate-A. The immune-cold cluster demonstrated a relatively bland immune-oncogenic gene expression profile. Color key: orange indicates high scores; blue indicates low scores. Scores are displayed on the same scale via a Z-transformation. **(b)** The proportion of immune cells (CD8+ cytotoxic T-cells, CD15+ neutrophils, CD68+ macrophages, FOXP3+ T-reg cells) relative to ERG+ tumor cells directly correlated with the immune clusters (n = 11 immune-hot and n = 5 immune-cold cases) inferred from NanoString transcriptomic profiling (p = 0.0201). Box-plots are represented by median and lower to upper quartile values, and the vertical line extends from the minimum to the maximum value. **(c)** As inferred using NanoString gene expression data, head and neck tumors were relatively enriched for mast cells.

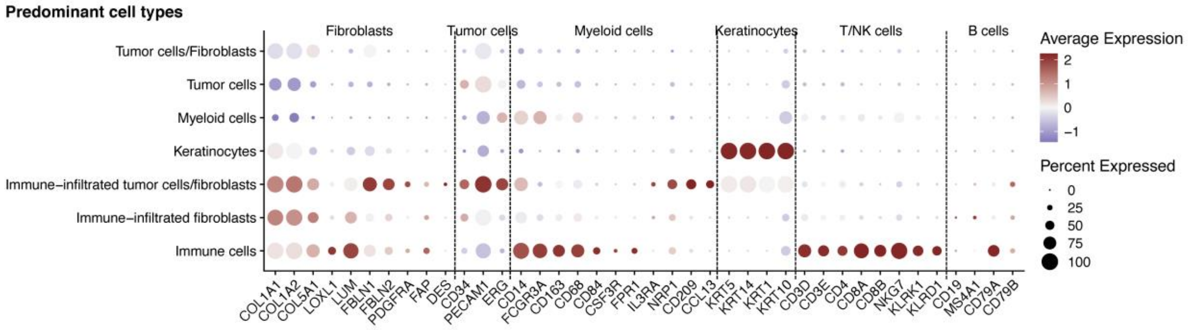

Supplementary Figure 3. Dot plot of spatial datasets showing gene expression patterns of cell type-specific markers. The dot size represents the percentage of cells expressed in each cell type, and the color intensity represents the average scaled expression relative to all cell types.
