# Supplementary material for: Spatial transcriptomics reveal topological immune landscapes of Asian head and neck angiosarcoma
Source: Commun Biol. 2023 Apr 27;6:461. doi: 10.1038/s42003-023-04856-5 (PMC10140281; doi:10.1038/s42003-023-04856-5)
Supplement: Supplementary file 3 — Description of Additional Supplementary Files [file 42003_2023_4856_MOESM3_ESM.pdf]

Description of Additional Supplementary Files

File name: Supplementary Data 1

Description: Source data behind the graphs in the paper

File name: Supplementary Data 2

Description: Source data behind the graphs in the paper

File name: Supplementary Data 3

Description: Source data behind the graphs in the paper

File name: Supplementary Data 4

Description: Source data behind the graphs in the paper

File name: Supplementary Data 5

Description: Source data behind the graphs in the paper
